# From “synthetic” to defined microbial communities for clearer terminology

**DOI:** 10.1038/s41467-026-74251-1

**Published:** 2026-06-08

**Authors:** Hanna Koch, Thomas Clavel, Cintia Mayr, Benjamin L. Coltman, Michael Schloter, Julia A. Vorholt, Yolanda Sanz, Tomislav Cernava, Gwyn A. Beattie, Lene Lange, Stéphane Chaillou, Ákos T. Kovács, Hauke Smidt, Corné M. J. Pieterse, Tanja Kostic, Omri M. Finkel, Christopher E. Lawson, Luca Cocolin, Jesús Mercado-Blanco, Robert D. Finn, Kalliope K. Papadopoulou, Matthew Ryan, Marco Candela, Paul D. Cotter, Gabriele Berg, Orla O’Sullivan, Manuel Delgado-Baquerizo, Pankaj Trivedi, Trevor C. Charles, Brajesh K. Singh, Günter Brader, Malek Marian, Angela Sessitsch

**Affiliations:** 1 Bioresources Unit, Center for Health & Bioresources, AIT Austrian Institute of Technology GmbH, Tulln an der Donau, Austria; 2 Division of Microbial Ecology, Centre for Microbiology and Environmental Systems Science, University of Vienna, Vienna, Austria; 3 Functional Microbiome Research Group, Institute of Medical Microbiology, RWTH University Hospital, Aachen, Germany; 4 Comparative Microbiome Analysis, Helmholtz, Oberschleissheim, Germany; 5 Institute of Microbiology, ETH Zurich, Zurich, Switzerland; 6 Institute of Agrochemistry and Food Technology, National Research Council (IATA-CSIC), Paterna-Valencia, Spain; 7 Faculty of Environmental and Life Sciences, School of Biological Sciences, University of Southampton, Southampton, UK; 8 Department of Plant Pathology, Entomology & Microbiology, Iowa State University, Ames, IA USA; 9 LL-BioEconomy, Research & Advisory, Valby, Denmark; 10 KMUTT, King Mongkut’s University of Technology, Bangkok, Thailand; 11 Université Paris-Saclay, INRAE, UMR1319 Institut MICALIS, Jouy-en-Josas, France; 12 Institute of Biology, Leiden University, Leiden, The Netherlands; 13 Laboratory of Microbiology, Wageningen University & Research, Wageningen, The Netherlands; 14 UNLOCK, Wageningen University & Research and Delft University of Technology, Wageningen and Delft, The Netherlands; 15 Plant-Microbe Interactions, Department of Biology, Utrecht University, Utrecht, The Netherlands; 16 Department of Plant & Environmental Sciences, The Alexander Silberman Institute of Life Sciences, The Hebrew University of Jerusalem, Jerusalem, Israel; 17 Department of Chemical Engineering & Applied Chemistry, University of Toronto, Toronto, ON Canada; 18 Department of Agriculture, Forest and Food Sciences, University of Turin, Grugliasco, Torino Italy; 19 Microbiology of Agroforestry Ecosystems, Department Soil and Plant Microbiology, Estación Experimental del Zaidín, Consejo Superior de Investigaciones Científicas (EEZ-CSIC), Granada, Spain; 20 European Molecular Biology Laboratory, European Bioinformatics Institute (EMBL-EBI), Wellcome Trust Genome Campus, Hinxton, Cambridge UK; 21 Department of Biochemistry and Biotechnology, Laboratory of Plant and Environmental Biotechnology, University of Thessaly, Larissa, Greece; 22 CABI, Ascot, Berkshire, UK; 23 Unit of Microbiome Science and Biotechnology, Department of Pharmacy and Biotechnology (FaBiT), Alma Mater Studiorum - University of Bologna, Bologna, Italy; 24 Teagasc Food Research Centre, Cork, Ireland; 25 APC Microbiome, Cork, Ireland; 26 VistaMilk, Cork, Ireland; 27 Institute of Environmental Biotechnology, Graz University of Technology, Graz, Austria; 28 Leibniz Institute for Agricultural Engineering and Bioeconomy (ATB), Potsdam, Germany; 29 Laboratorio de Biodiversidad y Funcionamiento Ecosistémico, Instituto de Recursos Naturales y Agrobiología de Sevilla (IRNAS), CSIC, Sevilla, Spain; 30 Department of Plant and Soil Science, Institute of Genomics for Crop Abiotic Stress Tolerance (IGCAST), Texas Tech University, Lubbock, TX USA; 31 Department of Biology, University of Waterloo, Waterloo, Ontario Canada; 32 School of Agriculture and Environment, The UWA Institute of Agriculture, University of Western Australia, Perth, WA Australia

**Keywords:** Microbiome, Microbial ecology, Clinical microbiology, Applied microbiology

## Abstract

Consortia of microbial isolates, also known as synthetic communities (SynComs), are increasingly used to study and harness microbe-microbe and microbe-host interactions. Since “synthetic” potentially evokes negative connotations, we propose adopting the term “Defined Microbial Community” for practical applications.

Microbiomes are increasingly studied to drive medical, environmental, and biotechnological innovations. Understanding their functions and how to modulate them is a powerful tool in tackling global challenges related to food safety and security, climate change, ecological restoration, as well as animal and human health. In recent years, microbiome research and applications have undergone a conceptual transition. Early strategies focused on the application of single strains to modulate host or environmental outcomes. However, the limited ecological stability and efficacy of these approaches have prompted a shift towards more complex, yet defined, microbial consortia that better capture the taxonomic and functional microbiome diversity^1^ (Box 1). For example, the application of defined root-associated consortia to *Arabidopsis thaliana* and crop plants were shown to improve resilience and promote growth under biotic and abiotic stress^2,3^. Plant growth enhancing effects of multi-species consortia were also observed in field trials^4^. In humans, the shift from traditional probiotics to consortia-based formulations, also referred to as live biotherapeutics, marks a significant advancement in gut health^5^.

## Box 1

**Case studies on the application of microbial consortia in fundamental research and applied settings**

*Plant microbiome—fundamental research*

Carlström and colleagues conducted drop-out and late-introduction experiments by inoculating *Arabidopsis thaliana* with Defined Microbial Communities under gnotobiotic conditions (original publication: SynComs) derived from a resource of 62 native bacterial strains, testing how arrival order shapes community structure in the phyllosphere^27^. The study aimed to understand the assembly rules governing the establishment of plant microbiota and to determine the extent to which microbial community members interact, specifically examining how priority effects shape phyllosphere community structure. The results showed that community assembly is historically contingent and subject to priority effects, with missing strains able to invade an already established microbiota to varying degrees, while the established community as a whole remained largely resistant to and unaffected by latecomers.

System: *Arabidopsis thaliana* phyllosphere; scale gnotobiotic plant assays (axenic system with host and amended strains); complexity: 62 bacterial strains belonging to 42 genera; design: the strains are native phyllosphere isolates to capture the natural microbial diversity.

*Plant microbiome—microbial consortium application*

Fonseca-García and colleagues analyzed the impact of a Defined Microbial Community (original publication: SynCom) on the native rhizosphere community of sorghum as well as the host response to Defined Microbial Community application under both gnotobiotic and field conditions^28^. By this, the authors could evaluate strain growth patterns within the Defined Microbial Community under different growth conditions and effect of Defined Microbial Community application on the host phenotype with focus on potential drought stress resilience. The amendment of the Defined Microbial Community in the field impacted community composition without affecting the abundance patterns of its members within the native rhizosphere community. This community modulation impacted the plant transcriptome and resulted in an increase of plant biomass under normal irrigation condition.

System: *Sorghum bicolor* rhizosphere; scale: in vitro (no host), gnotobiotic plant assays (sterile system with host) and in the field (native community with host); complexity: 57 bacterial strains belonging to 16 genera; design: strains were selected either based on network analysis or based on their ability to use sorghum exudates for growth.

*Human microbiome—fundamental research*

Becker and colleagues designed a Defined Microbial Community of human gut bacteria (SIHUMI) to recapitulate the main functions of the whole ecosystem^29^. They developed an experimental model that enhances reproducibility between studies and can be used as a backbone community amendable with additional strains to functionally investigate microbe-microbe and microbe-host interactions. Gnotobiotic rats colonized with SIHUMI shared several features of conventionally colonized animals: short-chain fatty acid production, mucin degradation, bilirubin metabolism, and responses to diet. In subsequent studies, the model community was used to investigate effects on diet-induced obesity and intestinal inflammation.

System: human gut; scale: in vitro (no host) and gnotobiotic animals; complexity: 7 to 8 bacterial strains in the original model; design: strains were selected based on expert knowledge of their metabolism, interactions, and occurrence in the human gut.

*Human microbiome—microbial consortium application*

Louie and colleagues tested the effects of the Defined Microbial Community VE303 on the recurrence of *Clostridioides difficile* infection (CDI)^30^. The primary objective was to determine the recommended VE303 dosing for a phase 3 trial. High-dose application of VE303 significantly lowered recurrent CDI in high-risk adults. VE303 was well tolerated with mostly mild gastrointestinal adverse events, supporting progression to a larger phase 3 study to validate the results.

System: human gut; scale: in vivo, phase 2, randomized, double-blind, placebo-controlled, dose-ranging trial; complexity: 8 strains mostly *Clostridium* clusters IV, XIVa, and XVII; design: The strains were isolated from healthy human donors and tested in different mixtures in the cefoperazone mouse model of CDI.

Intentionally assembled microbial communities are often referred to as synthetic communities (SynComs). Their design and use for studying community dynamics represent a convergence of ecological theory, systems biology, and translational research. As model systems, they enable precise investigations of community dynamics and metabolic interactions, paving the way for robust and reproducible microbiome interventions. However, the term is used inconsistently across disciplines, creating confusion within and between research fields. This is partly due to the term’s semantic association with synthetic biology, even though its use most often refers to consortia of naturally occurring, non-genetically modified microorganisms in host-microbiome and environmental systems. Rightly or wrongly, this inconsistency may contribute to public skepticism toward microbiome applications and hinder their use in medicine, food biotechnology, sustainable agriculture and environmental applications. A clearer and more representative terminology is therefore needed to support effective communication and broader societal uptake of microbiome-based products.

## SynComs to test causal relationships in microbiomes

The term “synthetic” was originally introduced in molecular biology to describe a cell constructed de novo in the laboratory. Over time, its meaning broadened with the establishment of the field of synthetic biology encompassing a wide range of microbial engineering efforts, including organisms designed for biomanufacturing or bioprocessing. One of the earliest applications of the term “synthetic” to microbial communities was in 2006, in a study assessing how sampling size affects taxa–area relationships^6^. In 2008, Kim et al. introduced the term “synthetic community”, defining it as a deliberately designed, artificial community, without implying that the bacterial species co-occur in nature^7^. The prefix “Syn”- in SynCom historically derives from the word synthetic, but is linked to the Greek prefix “syn”, meaning “together” or “united”. From this perspective, SynComs can be framed within synthetic ecology, which involves the rational design and targeted manipulation of microbial community composition. These communities, with reduced complexity and increased controllability, allow researchers to study the dynamics and functions of microbial communities^8^. This differs from synthetic biology, which typically involves the genetic modification of microorganisms^9^.

**Fig. 1:**
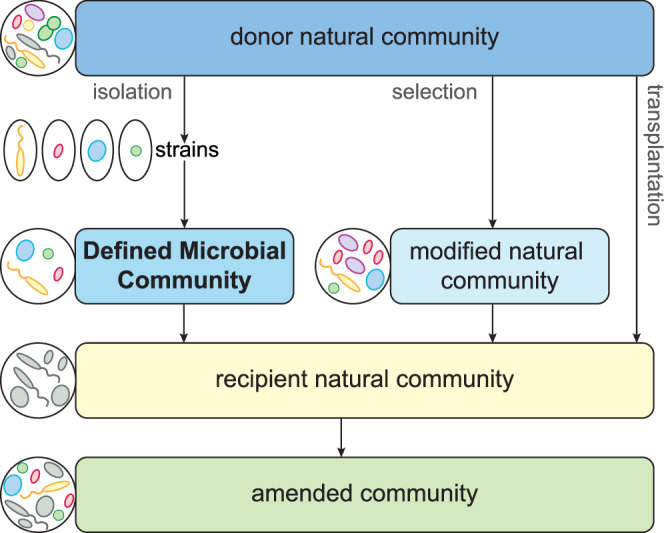
Overview of microbiome-based amendments to modulate the function and/or composition of a recipient natural community. Shown from left to right: inoculation with a Defined Microbial Community, a modified natural community, or whole community transplant. While the strains chosen for a Defined Microbial Community may not originate from the same natural donor community, the composition should be ecologically informed and supported by evidence on interactions and stability.

In recent years, interest in SynComs has grown rapidly. Numerous review articles cover their application in different systems^10–12^, and general aspects^13^ including underlying ecological principles^14^ and best practices for design^15,16^. However, the criteria used to define what a SynCom is vary across studies. The term SynCom has been used to described intentionally assembled communities of cultured strains (bottom-up design)^11,13,15^. Alternatively, it refers to communities shaped through ecological pressures or host selection that reduce the complexity of naturally occurring microbial communities (top-down design)^11^; we refer to these as “modified natural communities” (Fig. 1). Furthermore, the term SynCom has evolved in its usage. Originally intended to describe communities used to study microbial interactions, or host-microbe interactions, it now also refers to multi-strain applications such as probiotics and biopesticides^17^. This expansion in usage raises questions about public acceptance of the term SynCom. The semantic association with genetic engineering and synthetic biology contrasts with the way the scientific community often uses the term SynCom to describe a consortium of non-genetically modified microorganisms derived from natural sources. Moreover, the term “synthetic” is inherently ambiguous and often carries negative connotations due to a common preference for “natural” over “synthetic”, described as natural-is-better or naturalness bias^18^. We therefore recommend avoiding the term “synthetic” in the context of microbial applications.

## Defined microbial communities for microbiome modulation

The intentional modulation of microbiomes to perform desired functions is referred to as microbiome engineering. Typically, this process is guided by fundamental scientific principles and quantitative design to create microbiomes that perform desired functions^19^. Microbiome engineering represents an intentional alteration of microbial communities using direct or indirect processes, or a combination of both. Examples of indirect processes include host dietary intervention and application of exogenous compounds (e.g., prebiotics). Whereas, direct approaches include the introduction of single microorganisms, defined microbial communities, modified natural communities, and direct microbiome transplantation into recipient microbial communities. In the context of microbiome engineering, multi-strain inoculants are sometimes called “SynComs”^20^.

Here, we propose using the term “Defined Microbial Community” for consortia consisting of multiple cultured and well-described microbial strains that are deliberately assembled in the laboratory (Fig. 1). The use of this term as an alternative to “SynCom” is not new and has been primarily, though not exclusively, used in medical microbiology^21,22^. Defined Microbial Communities are produced by intentionally combining known microbial strains, which should be part of well-characterized collections of taxonomically classified strains, preserved for long-term use. The community design may be guided by the functional and ecological properties of its members in the system of interest, ensuring compatibility, successful establishment, and performance within the target environment. Defined Microbial Communities may or may not contain genetically modified microorganisms, although the inclusion of the latter may incur regulatory approval requirements that can delay or restrict their use and application. In contrast to Defined Microbial Communities, microbiome-based applications involve introducing modified natural communities or whole community transplants (e.g., soil or fecal) to functionally improve a recipient microbiome (Fig. 1). These modified natural communities are not intentionally designed and assembled in the laboratory but rather arise from processes such as cell-size fractionation and enrichment based on metabolic properties. They represent a subset of a natural donor community and include enrichment cultures, or undefined starter cultures, which may contain as-yet uncultured microorganisms. Although the composition of these modified natural communities is not fully controlled, they should be thoroughly characterized before use^23^ and their natural donor communities should be selected on evidence-based criteria. Modified natural communities may lack reproducibility and standardization across experiments or applications because they are not deliberately assembled.

## Further considerations

A SynCom is often defined as a “synthetic community”, however the use and interpretation of the term vary across research fields, underscoring the potential benefits of aligning terminology and guidelines for reporting. Achieving a consensus on scientific concepts and terminology for microbial communities intended as regulatory-approved products is essential to ensure consistency across existing regulatory paths, which differ for genetically modified and non-modified microorganisms. More precise and unambiguous definitions will make it easier to navigate approval processes within diverse legislative frameworks. Although regulatory frameworks for various microbial consortia applications are still evolving^24^, multi-species products can be approved similarly to single-strain products if their composition is clearly defined. For example, the regulatory frameworks for live biotherapeutic products include both single strains and microbial consortia as recently summarized^25^. In the EU, the regulation for microbial plant biostimulants (EU Regulation 2019/1009)^26^ allows principally the use of “consortia of microorganisms”. The proposed term “Defined Microbial Community” aligns well with this regulatory language, but emphasizes “defined” to indicate a specified, reproducible composition of intentionally assembled, well-characterized strains. Additionally, the term avoids negative connotations and thus might help accelerate the implementation of microbiome-based solutions in human medicine, agriculture, food production, and environmental management.
